# CT appearance and measurements of the normal thyroid gland in goats

**DOI:** 10.1186/s12917-021-03047-w

**Published:** 2021-10-26

**Authors:** Filip Pankowski, Bartłomiej Jan Bartyzel, Sławomir Paśko, Agata Moroz, Marcin Mickiewicz, Olga Szaluś-Jordanow, Joanna Bonecka

**Affiliations:** 1 Department of Morphological Sciences, Institute of Veterinary Medicine, Warsaw University of Life Sciences-SGGW, Warsaw, Poland; 2 Virtual Reality Techniques Division, Institute of Micromechanics and Photonics, Faculty of Mechatronics, Warsaw University of Technology, Warsaw, Poland; 3 Division of Veterinary Epidemiology and Economics, Institute of Veterinary Medicine, Warsaw University of Life Sciences-SGGW, Warsaw, Poland; 4 Department of Small Animal Diseases with Clinic, Institute of Veterinary Medicine, Warsaw University of Life Sciences-SGGW, Warsaw, Poland

**Keywords:** Goats, Caprine, Thyroid gland, Parathyroid gland, CT

## Abstract

**Background:**

Goats are increasingly being kept as companion animals, thus their owners expect advanced medical care, including modern diagnostic imaging. Computed tomography (CT) is now widely used in veterinary medicine, in both clinical practice and for scientific purposes. So far, the CT appearance of various body parts has been described in goats, but reports on thyroid gland CT are still lacking. The thyroid gland in goats may become enlarged due to dietary, genetic or neoplastic disorders. CT examination, as in other animals and humans, could aid in the diagnosis of thyroid diseases in goats and could be used for research purposes. The aim of the study was to present the CT characteristics of the normal caprine thyroid gland, in particular its dimensions, volume and density.

**Results:**

Fifty-seven goats were included in the study. None of the animals had clinical, CT, post-mortem or histopathologic abnormalities in the thyroid gland. CT features of the thyroid gland were determined, such as dimensions, volume, density, location and shape. The presence of the thyroid isthmus and ectopic thyroid tissue was also assessed. The gland was visible in every animal as two homogenous, highly attenuating, well-circumscribed lobes located in the most cranial part of the trachea. The mean dimensions of the thyroid lobe were 30.3 × 12.7 × 6.7 mm, the mean density was 80.9 Hounsfield Units (HU) and the mean volume was 1.32 cm^3^ or 1.39 cm^3^, depending on the method used. Also, the internal parathyroid glands were visible in some animals.

**Conclusions:**

For the first time, the normal CT appearance of the thyroid gland has been presented. CT clearly shows the thyroid gland in goats and therefore can be used in clinical practice and for research. The results of the current study may serve as a radiological guideline for practitioners and may be the basis for further CT studies on normal and diseased caprine thyroid glands.

**Supplementary Information:**

The online version contains supplementary material available at 10.1186/s12917-021-03047-w.

## Background

The growing use of computed tomography (CT) in veterinary medicine has been observed for years. In addition to its wide application in clinical practice, this modality is also used in research, especially in the field of veterinary anatomy. Normal CT appearance of selected body regions and anatomical systems has been described for different animal species [1, 2].

Goats are increasingly being kept as companion animals and are taken great care of by their owners, therefore there is a rising need for diagnostic imaging examinations in this species, including CT. To correctly describe and interpret CT scans, the observer must be familiar with the normal features of the examined area. Some body regions have been described in CT studies of goats. The computed tomographic appearance of a thorax was reported, where parameters such as its height and width, main blood vessels and bronchi diameter, vertebral heart score and lung densities were assessed [3]. The work additionally indicated the effectiveness of CT in diagnosing pulmonary diseases. Similar studies were performed on the caprine abdominal cavity, where the tomographic anatomy, organ dimensions, volume and densities were presented [4]. The CT characterization of the head, mammary gland, orbital cavity and eyes were also described [5–8]. In single case reports, CT was helpful in diagnosing diseases such as leukoencephalomyelitis, a giant cell tumor of the mandible, a brain abscess and sinusitis [9–12].

The thyroid gland in goats consists of two lobes located on each lateral surface of the cranial trachea, which are connected in their caudal end by a thin, poorly developed fibrous isthmus. The lobe is pear-shaped, fusiform or oval, and its mean gross dimension is 32.0 × 12.6 × 6.8 mm [13]. The gland may become enlarged (so-called goitre) due to dietary, genetic, and less commonly neoplastic disorders [14, 15]. A computed tomographic examination could aid in diagnosis of thyroid disease or in determination of the thyroid origin of the lesion, when localized swelling in the neck is present. However, to the authors’ knowledge, any reports of CT examinations of the thyroid gland in goats do not appear in existing literature. The CT appearance of a normal and diseased thyroid gland has already been described for dogs and cats [16–19].

The aim of the study was to present the CT appearance of the normal thyroid gland in goats, particularly the dimensions, volume, density and location of the gland. The obtained results may serve as a radiological guideline for clinicians and be the basis for further research, especially on the CT features of thyroid pathology.

## Results

Fifty-seven female goats from a dairy herd, belonging to the Polish White Improved and Polish Fawn Improved breeds, were included in the study. The mean age of the animals was 5.4 years (± 2.8 years), with a range from 1.6 to 13.1 years and the median 5.2 years.

**Fig. 1:**
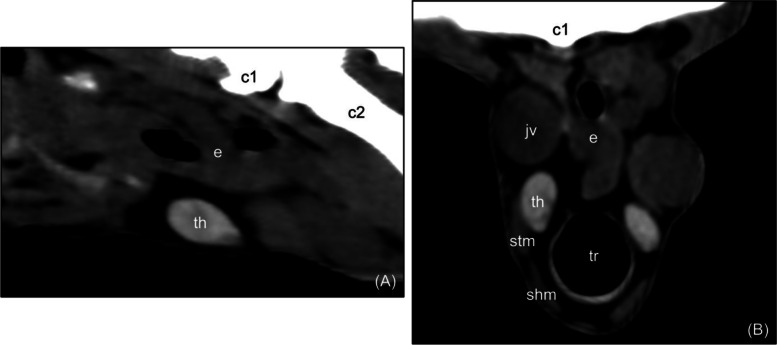
Computed tomographic image of a normal caprine thyroid gland in sagittal reconstruction (**A**) and transverse plane (**B**). The lobe is homodense and hyperdense compared to surrounding muscles and has smooth margins. WL = 80; WW = 220. th – thyroid gland; e – esophagus; c1 – first cervical vertebra; c2 – second cervical vertebra; jv – jugular vein; tr – trachea; stm – sternothyroid muscle; shm – sternohyoid muscle; WL – window level; WW – window width

The CT examination showed that the thyroid gland was clearly visible in all of the goats as two separate hyperdense lobes located on the lateral surfaces of the cranial part of the trachea. The lobes had a homogeneous attenuation and smooth margins and were surrounded by a variable amount of fat (Fig. 1). In some goats, the thyroid gland was isodense to the surrounding muscles, but even then, it was recognizable due to its characteristic shape and location. In the sagittal axis, the lobes were pear-shaped or oblong, while in the transverse axis, the lobes were triangular to oval. The mean dimensions of the lobe were 30.25 × 12.71 × 6.68 mm. The mean volume was 1.32 cm^3^ and 1.39 cm^3^, measured using the manual method and the ellipsoid formula, respectively. More data on the dimensions and volume is given in Table 1 and in Additional file 1. The mean thyroid density was 80.9 Hounsfield Units (HU). More data on thyroid density is provided in Table 2.

**Table 1:** Descriptive statistics of the normal thyroid gland dimensions and volume in goats

	Length [mm]	Width [mm]	Height [mm]	Volume (manual) [cm^3^]	Volume (formula) [cm^3^]
Minimum	16.70	8.70	4.40	0.40	0.46
Maximum	49.40	19.60	9.00	3.19	3.58
Range	32.70	10.90	4.60	2.79	3.12
Mean ± SD	30.25 ± 5.67	12.71 ± 1.94	6.68 ± 1.05	1.32 ± 0.54	1.39 ± 0.54
Median	29.75	12.70	6.70	1.22	1.31

**Table 2:** Descriptive statistics of the normal thyroid gland density in goats

	Density – cranial part [HU]	Density – middle part [HU]	Density – caudal part [HU]	Density – overall [HU]
Minimum	35.0	40.0	35.0	35.0
Maximum	129.0	140.0	133.0	140.0
Range	94.0	100.0	98.0	105.0
Mean ± SD	83.6 ± 21.1	80.3 ± 21.3	78.9 ± 20.8	80.9 ± 21.0
Median	83.0	80.0	78.5	80.0

HU – Hounsfield Units

An isthmus and thyroid ectopic tissue was not visible in any of the animals. The most cranial part of the thyroid gland was located just behind the cricoid cartilage of the larynx. The caudal range of the thyroid gland in relation to the tracheal rings was variable, as shown in Table 3.

**Table 3:** Descriptive statistics of the caudal range of a normal goat thyroid gland in relation to tracheal rings

	Minimum	Maximum	Range	Q25	Q50	Q75
Tracheal ring	3	7	4	5	5	5

**Fig. 2:**
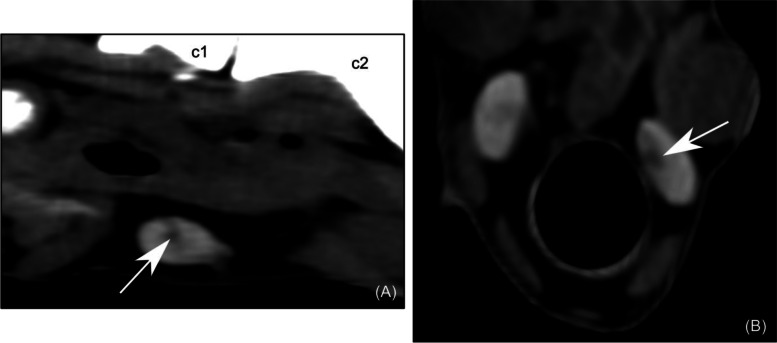
Computed tomographic image of a normal caprine internal parathyroid gland in a sagittal reconstruction (**A**) and transverse plane (**B**). The arrow shows the internal parathyroid gland, which is hypodense, poorly defined and located inside the cranial part of the thyroid lobe, close to its medial surface. WL = 80; WW = 220. c1 – first cervical vertebra; c2 – second cervical vertebra; WL –window level; WW – window width

A hypodense region of several millimeters, poorly defined, with indistinct margins, was visible in some animals in the cranial part of the thyroid lobe, close to its medial surface (Fig. 2). The location, shape and size of this region were consistent with the internal parathyroid gland visible on post-mortem and histologic examination.

Statistical analysis did not show any statistical differences between the parameters of the right and left thyroid lobe (p = 0.05). Correlation between the various parameters of the thyroid gland is shown in Additional file 2 and 3. The caudal range of the thyroid gland correlated only with the thyroid length, r = 0.23. The length correlated, for example, with the width, but not so much that this dependence was transferred to the caudal range of the thyroid gland. Considering the differentiation between the lobes, there was no correlation between the width and density for the right lobe, and no correlation between the height and length for the left lobe. There was an inverse correlation between the age of the goat and caudal range of the thyroid gland, while the volume of the thyroid gland correlated directly proportional with the goat’s age. Statistically, the thyroid density did not change with age and the greater the density of the gland, the greater was its volume. For example, the coefficient factor for the cranial part without the differentiation for the right and left lobe was 0.47.

All of the thyroid glands were normal on post-mortem and histologic examination.

## Discussion

As far as the authors know, this is the first study on the normal CT appearance of the thyroid gland in goats. It may be helpful for the clinicians describing thyroid CT scans and it complements the knowledge about CT characteristics of the thyroid gland in another animal species.

The thyroid gland was clearly visible on CT in every goat, which is consistent with the observations made in other animal species and humans. This is due to high content of iodine in the gland and therefore its higher attenuation than the surrounding soft tissues [20, 21].

In dogs, the CT features of thyroid neoplasia, including carcinoma, were described [22]. Carcinoma was also reported in caprine thyroid gland on histopathology [15]. In the rare case of an adult cat’s goiter, which was caused by diffuse follicular hyperplasia, the thyroid lobes and isthmus were enlarged, homogeneous in structure and isodense to the surrounding muscles on CT [23]. In humans, CT is especially important in preoperative planning to assess the extent of thyroid disease, invasion to the surrounding structures, lymph node metastases and also in staging and in searching for recurrent disease [24]. Due to the above and the fact that the thyroid gland can also be affected by various diseases in goats, CT examination can be used in suspected thyroid pathology in this species, especially when diffuse enlargement of the gland or nodular lesions are present.

The mean thyroid density in goats in the current study was 80.9 HU, which is much less compared to cats and dogs, 123.2 HU and 107.5 HU, respectively. These values could be influenced by differences in certain species and also by various methods of measuring the density. In the present study, due to the size of the thyroid gland, it was possible to create three regions of interest (ROI) of 10 mm^2^ each and place them in three different parts of the sagittal axis of each thyroid lobe. In the study on cats, however, the single ROI measuring 1 mm^2^ was placed on the transverse axis of the lobe on each slice containing the thyroid tissue, and in the dog study, the ROI was manually drawn around each transverse section of the thyroid lobe [16, 18].

The CT examination in goats may also be of potential use in the diagnosis of thyroid diseases that are associated with changes in the thyroid density. In humans, the density may decrease in thyroid diseases. It also reflects the functional status of the thyroid gland determined by the serum thyroid stimulating hormone (TSH) level, i.e. at low density values, TSH levels are too low (hyperthyroid state) or too high (hypothyroid state) [25]. In cats, however, no significant differences in thyroid density were found in those with normally functioning thyroid gland, having serum thyroxine (T4) levels within the reference range, and cats with hyperthyroidism, having serum T4 levels above normal [26]. Recent studies on dogs found that brachycephalic breeds had significantly lower thyroid HU values ​​than non-brachycephalic, and there was no significant correlation between HU values ​​and serum thyroid hormone levels in that group. Additionally, dogs with decreased density of the thyroid gland could also have normal levels of thyroid hormones [27]. It remains to be determined how the thyroid density changes in the diseased gland in goats and if there is a correlation between the density of the thyroid and its functional status in this species.

The caudal range of the thyroid gland in relation to the tracheal rings in the current study was in the range of 3–7 (the number corresponds to the number of the tracheal ring). It was similar to the range found in dogs, which was 4–8 [18]. The position of the caudal end of the thyroid gland outside this range may indicate a displacement of the thyroid gland by a lesion in the surrounding tissue or enlargement of the gland itself.

Measuring the volume of the thyroid gland with the use of ellipsoid formula and the method of manual drawing gave similar results. To obtain the most precise measurement, the manual method is suggested. However, for general clinical applications, when the most accurate dimensions of the thyroid gland are not essential, the much faster and straightforward ellipsoid formula method is adequate.

There is little information about the CT appearance of parathyroid glands in animals. In studies on dogs and cats from several years ago, these glands were not visible [16, 18]. In the most recent study, the parathyroid glands were variably visible in dogs and their visibility was generally described as poor. Contrast-enhanced scans did not show more glands compared to plain CT. The authors of that study suggest, that the reported glands’ dimensions should be treated with caution due to the difficulties in precisely defining their margins [28]. In the current study, similarly, the parathyroid glands were not always visible and their ill-defined margins limited precise measurements. Thus, it should be considered that internal parathyroid glands may be visible on a thyroid CT scan in goats and they must not be confused with a pathological lesion. In human medicine, plain CT alone is never used for imaging the parathyroid glands. Ultrasonography, scintigraphy using 99mTc-MIBI, four-dimensional computed tomography (4D CT), magnetic resonance imaging and recently a promising positron emission tomography (PET) with radiolabeled choline are used, especially in primary hyperparathyroidism, when diseased glands must be precisely located prior to minimally invasive parathyroidectomy [29].

The study has several limitations. First, there was no data on thyroid hormones level, which made it impossible to correlate them with thyroid density. However, the level of thyroid hormones in goats is influenced by many internal, environmental and nutritional factors, and the hormone values reported by various authors vary widely due to differences between the examined animals, conditions and measurement methods [30–32]. Therefore, even if the information on thyroid hormone level was given and related to the thyroid density, it might not be relevant for goats studied under different conditions. Secondly, the current study was performed on animals having clinical form of caprine arthritis-encephalitis (CAE). To the best of the authors’ knowledge, this disease does not cause anatomopathological lesions in the thyroid gland, nor does it affect its functioning. Thirdly, only female goats were used in the study. This is due to the fact, that in Poland goats are predominantly used to produce dairy products and females constitute the vast majority of the goat population. Therefore, the data given, especially the dimensions and volume of the gland, should be carefully applied to males. Finally, the contrast-enhanced scans were not performed, because these were postmortem examinations and the lack of blood flow would make it impossible for the contrast agent to reach the thyroid gland. Further studies are needed to determine how the image and density of the thyroid gland would change after administration of the intravenous contrast agent and whether it would improve the visibility of the parathyroid glands.

## Conclusions

For the first time, the CT appearance and measurements of the normal thyroid gland in goats were presented. This supplements the knowledge of CT anatomy of this gland in another animal species. The results of this study can be used as a reference when performing a thyroid CT scan in goats and can be the basis for further investigation of abnormalities in the caprine thyroid gland.

## Methods

### Animals

Initially, 70 goats with no clinical signs of thyroid disease were enrolled in the study. From this group, 57 goats were selected that did not show any abnormalities in the thyroid gland visible on CT, necropsy or histopathology. They were all female and belonged to the Polish White Improved and Polish Fawn Improved breeds and the mean age of the animals was 5.4 years (± 2.8 years), with a range from 1.6 to 13.1 years and the median of 5.2 years.

All of the goats were from a dairy herd and were sent for slaughter due to the clinical form of CAE, which was confirmed by ELISA (ID Screen MVV/CAEV Indirect Screening test, ID.vet; Innovative Diagnostics, Grabels, France). Euthanasia was performed by overdosing pentobarbital (Morbital, Biowet Puławy, Poland) administered intravenously at a dose of 30 mg/kg under general anesthesia, which was induced by intravenous administration of a mixture of xylazine (Xylapan, Vetoquinol, Poland) at a dose of 0.05 mg/kg and ketamine (VetAgroam, Poland) at a dose of 10 mg/kg.

## CT examination and image analysis

CT examination was performed immediately after euthanasia using a 16-row scanner (Philips & Neusoft Medical Systems) with parameters 120 kV, 150 mA, slice thickness 0.75 mm or 2 mm and reconstructed in a soft tissue algorithm. The area from the laryngeal inlet to the thoracic inlet was scanned. A DICOM viewer (Horos, Nimble Co LLC d/b/a Purview, Annapolis, MD, USA) was used for image analysis. Different windows were used to view CT images, depending on the structure analysed. Mostly, a window level of 80 and window width of 220 were the most beneficial.

**Fig. 3:**
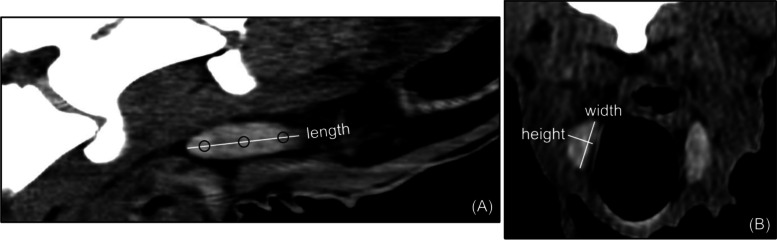
Computed tomographic image of the normal caprine thyroid lobe in sagittal reconstruction (**A**) and transverse plane (**B**). The lines show how the maximum length, width and height of the lobe were measured. The circles show how the thyroid density was measured. They represent ROI, which were placed in cranial, central and caudal part of the lobe. ROI – region of interest

**Fig. 4:**
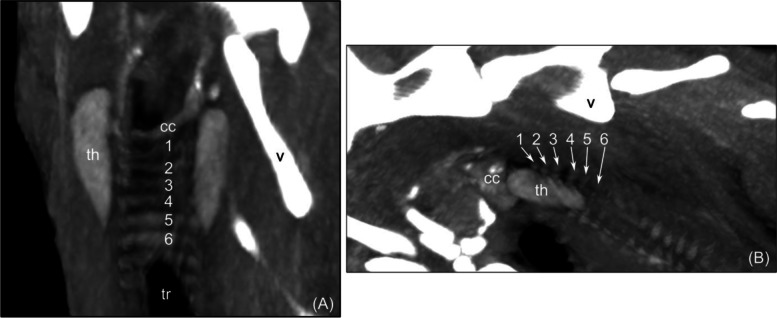
Computed tomographic maximum intensity projection (MIP) of the thyroid gland in the dorsal (**A**) and sagittal (**B**) planes. A method of measuring the caudal range of the thyroid gland in relation to tracheal rings is shown. th – thyroid, cc – cricoid cartilage, v – vertebra, tr – tracheal lumen, 1-6 – tracheal ring number

In multiplanar reconstruction (MPR), the tomographic characteristics of the thyroid gland, such as location, shape, structure, margins and density were determined. In the sagittal axis, the maximum length of both lobes was measured, and in the transverse axis, the maximum width and height. Additionally, in the sagittal axis, the thyroid density was assessed by placing three round ROI of 10 mm^2^ each, one at the cranial, central and caudal part of both lobes (Fig. 3). Next, the tomographic volume of the thyroid gland was measured by two methods. In the first method, the margins of both lobes were manually outlined on each transverse slice, creating multiple ROI. Then, based on the created ROI, a three-dimensional model and ROI volume were computed. In the second method, the ellipsoid formula was used ([length * width * height] π/6), based on the previously acquired measurements of the maximum length, width and height of the gland. Next, the thyroid isthmus and the thyroid ectopic tissue were searched for in the entire examined area. Finally, the caudal range of the thyroid gland relative to the tracheal rings was determined using maximum intensity projection (MIP) imaging (Fig. 4).

## Post-mortem and histologic examination

Post-mortem examination was performed immediately after CT examination. The thyroid gland was dissected, removed and cleaned of surrounding soft tissues. Each lobe was assessed macroscopically, and then placed in 10 % buffered formalin for a week. After this time, a histological examination was performed in the routine manner by tissue dehydration, paraffin embedding, 5 μm sectioning and staining with hematoxylin and eosin.

### Statistical analysis

Statistical calculations were performed in the R environment, version 4.0.3. One of the basic packages of this environment is „stats”. It includes a function that carries out the Shapiro-Wilk distribution normality test. Continuous distributions were compared with each other using the Pearson or Spearman correlation included in the „Hmisc” 4.5.0 package. Which correlation was used depended on the result of the Shapiro-Wilk test. Correlations between continuous and categorical variables were performed using ANOVA with the use of functions from the „stats” package. The Wilcoxon test was used to check whether there was a statistical difference between the corresponding parameters of the right and left thyroid lobe. The function that executes it was also part of the “stats” package.

## Supplementary Information

**Additional file 1.** Computed tomographic dimensions and volume of a normal goat thyroid lobes.

**Additional file 2.** Correlation between thyroid parameters with the differentiation into the right and left lobe. The lack of correlation is marked in bold. r – correlation coefficient; p – significance level.

**Additional file 3.** Correlation between thyroid parameters without the differentiation into the right and left lobe. The lack of correlation is marked in bold. r – correlation coefficient; p – significance level.

## Data Availability

All data generated or analysed during this study are included in this published article [and its supplementary information files].

## References

[1] Saunders JH (2009). Computed tomography - spiralling towards diagnosis. J Small Anim Pract.

[2] Keane M, Paul E, Sturrock CJ, Rauch C, Rutland CS. Computed tomography in veterinary medicine: Currently published and tomorrow’s vision. In: Halefoglu AM, editor. Computed Tomography–Advanced Applications. InTechOpen. 2016. doi:10.5772/intechopen.68556.

[3] Ohlerth S, Becker-Birck M, Augsburger H, Jud R, Makara M, Braun U (2012). Computed tomography measurements of thoracic structures in 26 clinically normal goats. Res Vet Sci.

[4] Braun U, Irmer M, Augsburger H, Ohlerth S (2011). Computed tomography of the abdomen in Saanen goats: I. Reticulum, rumen and omasum. Schweiz Arch Tierheilkd.

[5] Tohidifar M, Goodarzi N, Masoudifard M (2020). Anatomy of the head in the Saanen goat: a computed tomographic and cross-sectional approach. Anat Sci Int.

[6] González-Romano N, Arencibia A, De Los Monteros AE, Rodríguez E, Rivero M, Vázquez JM (2000). Anatomical evaluation of the caprine mammary gland by computed tomography, radiology and histology. Anat Histol Embryol.

[7] Madkour N, Amin M, Karkoura A, Alsafy M, ElGendy S (2016). Computed Tomography and Gross Anatomical Studies of the Orbital Cavity of the Baladi Goat (*Capra hircus*). Alexandria J Vet Sci.

[8] Tusler CA, Good KL, Maggs DJ, Zwingenberger AL, Reilly CM (2017). Gross, histologic, and computed tomographic characterization of nonpathological intrascleral cartilage and bone in the domestic goat (*Capra aegagrus hircus*). Vet Ophthalmol.

[9] DeVilbiss B, Neelis D, Ochoa J, Ziegler J, Barrington G (2013). Computed tomography findings in a 5-year-old Australian Cashmere goat (*Capra hircus*) suffering leukoencephalomyelitis due to caprine arthritis encephalitis virus. Can Vet J.

[10] Dixon J, Weller R, Jeckel S, Pool R, Mcsloy A (2016). Imaging diagnosis–The computed tomographic appearance of a giant cell tumor affecting the mandible in a pygmy goat. Vet Radiol Ultrasound.

[11] Gerros TC, Mattoon JS, Snyder SP (1998). Use of computed tomography in the diagnosis of a cerebral abscess in a goat. Vet Radiol Ultrasound.

[12] Barrington GM, Tucker RL (1996). Use of computed tomography to diagnose sinusitis in a goat. Vet Radiol Ultrasound.

[13] Pankowski F, Paśko S, Bonecka J, Szaluś-Jordanow O, Mickiewicz M, Moroz A (2020). Ultrasonographic and anatomical examination of normal thyroid and internal parathyroid glands in goats. PLoS One.

[14] Smith MC, Sherman DM (2009). Goat Medicine.

[15] Löhr CV (2013). One hundred two tumors in 100 goats (1987–2011). Vet Pathol.

[16] Drost WT, Mattoon JS, Samii VF, Weisbrode SE, Hoshaw-Woodard SL (2004). Computed tomographic densitometry of normal feline thyroid glands. Vet Radiol Ultrasound.

[17] Drost WT, Mattoon JS, Weisbrode SE (2006). Use of helical computed tomography for measurement of thyroid glands in clinically normal cats. Am J Vet Res.

[18] Taeymans O, Schwarz T, Duchateau L, Barberet V, Gielen I, Haskins M (2008). Computed tomographic features of the normal canine thyroid gland. Vet Radiol Ultrasound.

[19] Bertolini G, Drigo M, Angeloni L, Caldin M (2017). Incidental and nonincidental canine thyroid tumors assessed by multidetector row computed tomography: a single-centre cross sectional study in 4520 dogs. Vet Radiol Ultrasound.

[20] Iida Y, Konishi J, Harioka T, Misaki T, Endo K, Torizuka K (1983). Thyroid CT number and its relationship to Iodine concentration. Radiology.

[21] Taeymans O, Peremans K, Saunders JH (2007). Thyroid imaging in the dog: Current status and future directions. J Vet Intern Med.

[22] Deitz K, Gilmour L, Wilke V, Riedesel E (2014). Computed tomographic appearance of canine thyroid tumours. J Small Anim Pract.

[23] Galgano M, Spalla I, Callegari C, Patruno M, Auriemma E, Zanna G (2014). Primary hypothyroidism and thyroid goiter in an adult cat. J Vet Intern Med.

[24] Traylor KS (2020). Computed tomography and MR imaging of thyroid disease. Radiol Clin North Am.

[25] Pandey V, Reis M, Zhou Y (2016). Correlation between computed tomography density and functional status of the thyroid gland. J Comput Assist Tomogr.

[26] Bush JL, Nemanic S, Gordon J, Bobe G (2017). Computed tomographic characteristics of the thyroid glands in eight hyperthyroid cats pre- and postmethimazole treatment compared with seven euthyroid cats. Vet Radiol Ultrasound.

[27] Amorós O, Espada Y, Vila A, Jiménez A, Novellas R (2021). Pre-contrast CT attenuation of the thyroid gland is lower in brachycephalic dogs versus non-brachycephalic dogs. Vet Radiol Ultrasound.

[28] Lautscham E, von Klopmann C, Schaub S, Stengel C, Hartmann A (2020). CT imaging features of the normal parathyroid gland in the dog. Tierarztl Prax Ausg K Kleintiere Heimtiere.

[29] Giovanella L, Bacigalupo L, Treglia G, Piccardo A (2021). Will ^18^F-fluorocholine PET/CT replace other methods of preoperative parathyroid imaging?. Endocrine.

[30] Todini L (2007). Thyroid hormones in small ruminants: Effects of endogenous, environmental and nutritional factors. Animal.

[31] Błaszczyk B, Udała J, Ga̧czarzewicz D (2004). Changes in estradiol, progesterone, melatonin, prolactin and thyroxine concentrations in blood plasma of goats following induced estrus in and outside the natural breeding season. Small Rumin Res.

[32] Riis P, Madsen A (1985). Thyroxine concentrations and secretion rates in relation to pregnancy, lactation and energy balance in goats. J. Endocrinol.

