# Supplementary material for: CT appearance and measurements of the normal thyroid gland in goats
Source: BMC Vet Res. 2021 Oct 26;17:337. doi: 10.1186/s12917-021-03047-w (PMC8547050; doi:10.1186/s12917-021-03047-w)
Supplement: Supplementary file 1 — Additional file 1. Computed tomographic dimensions and volume of a normal goat thyroid lobes. [file 12917_2021_3047_MOESM1_ESM.doc]

| No. | Left thyroid lobe | | | | | Right thyroid lobe | | | | |
| --- | --- | --- | --- | --- | --- | --- | --- | --- | --- | --- |
|  | Length  [mm] | Width  [mm] | Height  [mm] | Volume – manual [cm^3^] | Volume – formula [cm^3^] | Length  [mm] | Width  [mm] | Height  [mm] | Volume – manual [cm^3^] | Volume – formula [cm^3^] |
| 1 | 31,6 | 12,9 | 6,5 | 1,19 | 1,39 | 28,5 | 11,6 | 6,1 | 0,73 | 1,06 |
| 2 | 36,9 | 12,3 | 6,9 | 1,78 | 1,64 | 38,4 | 16,1 | 7,4 | 2,36 | 2,40 |
| 3 | 30,1 | 12,7 | 6,5 | 1,45 | 1,30 | 25,9 | 12,2 | 5,2 | 0,89 | 0,86 |
| 4 | 26,3 | 10,8 | 6,0 | 1 | 0,89 | 35,3 | 10,5 | 5,3 | 1,12 | 1,03 |
| 5 | 37,3 | 13,3 | 7,0 | 1,6 | 1,82 | 31,6 | 12,5 | 6,1 | 1,07 | 1,26 |
| 6 | 35,5 | 11,8 | 6,4 | 1,27 | 1,40 | 36,2 | 11,3 | 6,3 | 1,22 | 1,35 |
| 7 | 27,4 | 14,3 | 7,4 | 1,18 | 1,52 | 29,3 | 14,4 | 7,5 | 1,61 | 1,66 |
| 8 | 49,4 | 11,6 | 6,6 | 1,83 | 1,98 | 43,2 | 12,1 | 7,0 | 1,95 | 1,92 |
| 9 | 35,6 | 13,7 | 8,0 | 2,07 | 2,04 | 30,5 | 13,4 | 7,0 | 1,83 | 1,50 |
| 10 | 26,6 | 11,6 | 5,8 | 0,85 | 0,94 | 24,7 | 13,1 | 6,3 | 1,02 | 1,07 |
| 11 | 26,6 | 15,1 | 8,6 | 1,86 | 1,81 | 27,4 | 16,1 | 8,6 | 2 | 1,99 |
| 12 | 27,0 | 11,4 | 6,1 | 1,09 | 0,98 | 26,4 | 12,3 | 5,8 | 1,09 | 0,99 |
| 13 | 34,5 | 10,9 | 5,4 | 0,89 | 1,06 | 19,0 | 9,0 | 6,0 | 0,57 | 0,54 |
| 14 | 32,8 | 12,7 | 6,5 | 1,37 | 1,42 | 26,5 | 13,2 | 6,8 | 1,36 | 1,25 |
| 15 | 35,3 | 13,4 | 7,6 | 2,18 | 1,88 | 38,1 | 15,1 | 8,2 | 2,56 | 2,47 |
| 16 | 25,8 | 13,3 | 7,1 | 1,15 | 1,28 | 27,0 | 13,1 | 8,2 | 1,18 | 1,52 |
| 17 | 25,7 | 13,5 | 7,5 | 1,22 | 1,36 | 30,7 | 12,7 | 5,9 | 1,16 | 1,20 |
| 18 | 29,6 | 13,6 | 7,6 | 1,5 | 1,60 | 26,8 | 11,5 | 7,5 | 1,17 | 1,21 |
| 19 | 21,5 | 10,3 | 6,4 | 0,69 | 0,74 | 24,5 | 10,9 | 4,7 | 0,54 | 0,66 |
| 20 | 34,5 | 16,3 | 8,5 | 2,08 | 2,50 | 34,8 | 14,9 | 8,0 | 2,11 | 2,17 |
| 21 | 20,5 | 11,3 | 5,7 | 0,66 | 0,69 | 24,6 | 10,2 | 5,2 | 0,61 | 0,68 |
| 22 | 29,0 | 10,3 | 5,3 | 0,88 | 0,83 | 32,1 | 10,2 | 5,4 | 0,72 | 0,93 |
| 23 | 23,4 | 10,2 | 5,4 | 0,66 | 0,67 | 23,0 | 8,7 | 4,4 | 0,43 | 0,46 |
| 24 | 26,1 | 9,7 | 5,7 | 0,78 | 0,76 | 32,4 | 10,6 | 6,5 | 0,84 | 1,17 |
| 25 | 16,7 | 10,0 | 6,6 | 0,62 | 0,58 | 18,0 | 11,0 | 5,7 | 0,59 | 0,59 |
| 26 | 25,8 | 13,1 | 7,4 | 1,37 | 1,31 | 24,8 | 13,3 | 7,5 | 1,23 | 1,30 |
| 27 | 25,5 | 13,5 | 7,3 | 1,45 | 1,32 | 25,7 | 13,3 | 6,7 | 1,13 | 1,20 |
| 28 | 31,6 | 14,4 | 7,1 | 1,7 | 1,69 | 31,3 | 14,8 | 6,2 | 1,6 | 1,50 |
| 29 | 25,2 | 11,8 | 6,3 | 0,73 | 0,98 | 26,4 | 12,9 | 5,8 | 0,87 | 1,03 |
| 30 | 29,9 | 12,0 | 6,6 | 0,85 | 1,24 | 27,3 | 10,9 | 4,7 | 0,7 | 0,73 |
| 31 | 29,3 | 12,3 | 7,7 | 1,37 | 1,54 | 29,0 | 12,7 | 8,0 | 1,75 | 1,45 |
| 32 | 30,0 | 16,7 | 8,5 | 1,51 | 2,23 | 28,6 | 13,6 | 8,4 | 1,47 | 1,71 |
| 33 | 23,0 | 12,4 | 4,8 | 0,88 | 0,72 | 30,0 | 13,4 | 5,9 | 1 | 1,24 |
| 34 | 31,8 | 13,2 | 5,8 | 1 | 1,27 | 32,7 | 13,2 | 5,6 | 1,2 | 1,27 |
| 35 | 27,0 | 12,0 | 7,9 | 1 | 1,34 | 29,2 | 13,2 | 8,9 | 1,5 | 1,80 |
| 36 | 32,1 | 15,0 | 7,3 | 1,48 | 1,84 | 26,0 | 14,3 | 7,5 | 1,24 | 1,46 |
| 37 | 33,3 | 14,2 | 6,9 | 1,35 | 1,71 | 31,7 | 12,8 | 7,0 | 1,35 | 1,49 |
| 38 | 29,0 | 10,7 | 5,5 | 0,86 | 0,89 | 28,6 | 12,0 | 6,2 | 1 | 1,11 |
| 39 | 30,0 | 13,8 | 7,4 | 1,44 | 1,60 | 26,0 | 12,1 | 6,1 | 0,9 | 1,00 |
| 40 | 27,0 | 11,0 | 5,1 | 0,64 | 0,79 | 23,9 | 9,2 | 4,8 | 0,4 | 0,55 |
| 41 | 32,4 | 17,4 | 7,2 | 1,96 | 2,13 | 28,4 | 12,6 | 7,0 | 1,19 | 1,31 |
| 42 | 36,8 | 17,0 | 7,6 | 2,33 | 2,49 | 31,1 | 18,0 | 8,0 | 2,15 | 2,34 |
| 43 | 23,4 | 11,3 | 5,1 | 0,58 | 0,71 | 24,4 | 13,0 | 6,7 | 1,07 | 1,11 |
| 44 | 38,8 | 12,9 | 6,7 | 1,8 | 1,76 | 34,0 | 11,4 | 6,1 | 1,01 | 1,24 |
| 45 | 40,6 | 19,6 | 8,6 | 3,19 | 3,58 | 42,4 | 16,2 | 8,0 | 2,87 | 2,88 |
| 46 | 38,7 | 13,6 | 6,2 | 1,94 | 1,71 | 39,4 | 12,5 | 6,5 | 1,65 | 1,68 |
| 47 | 29,0 | 14,1 | 6,8 | 1,43 | 1,46 | 31,2 | 12,4 | 6,7 | 1,36 | 1,36 |
| 48 | 24,1 | 14,6 | 6,8 | 2,39 | 1,25 | 26,3 | 14,3 | 6,4 | 1,72 | 1,26 |
| 49 | 33,8 | 10,2 | 5,3 | 0,91 | 0,96 | 31,5 | 11,9 | 6,6 | 1,2 | 1,30 |
| 50 | 47,4 | 13,3 | 6,9 | 1,7 | 2,28 | 40,0 | 13,9 | 6,3 | 1,75 | 1,83 |
| 51 | 31,6 | 13,3 | 8,0 | 1,64 | 1,76 | 34,3 | 14,6 | 9,0 | 2,5 | 2,36 |
| 52 | 33,4 | 8,9 | 5,1 | 0,63 | 0,79 | 26,3 | 10,0 | 4,6 | 0,48 | 0,63 |
| 53 | 28,6 | 11,0 | 6,7 | 0,91 | 1,10 | 25,3 | 13,8 | 7,2 | 1,17 | 1,32 |
| 54 | 34,8 | 10,9 | 5,7 | 0,88 | 1,13 | 35,0 | 11,5 | 6,7 | 1 | 1,41 |
| 55 | 33,3 | 12,3 | 7,8 | 1,5 | 1,67 | 30,7 | 12,2 | 7,4 | 1,57 | 1,45 |
| 56 | 26,7 | 11,3 | 8,1 | 1,54 | 1,28 | 29,9 | 12,4 | 6,1 | 1,24 | 1,18 |
| 57 | 34,7 | 13,6 | 7,0 | 1,77 | 1,73 | 37,9 | 11,5 | 7,1 | 1,38 | 1,62 |
